# Preoperative cognitive-behavioural intervention improves in-hospital mobilisation and analgesic use for lumbar spinal fusion patients

**DOI:** 10.1186/s12891-016-1078-8

**Published:** 2016-05-20

**Authors:** Nanna Rolving, Claus Vinther Nielsen, Finn Bjarke Christensen, Randi Holm, Cody Eric Bünger, Lisa Gregersen Oestergaard

**Affiliations:** Diagnostic Centre, Regional Hospital Silkeborg, Falkevej 1-3, 8600 Silkeborg, Denmark; Regional Hospital Silkeborg, Silkeborg, Denmark; Department of Social Medicine and Rehabilitation, School of Public Health, Aarhus University, Aarhus, Denmark; Public Health and Quality Improvement, Central Denmark Region, Aarhus, Denmark; Department of Clinical Medicine, Aarhus University Hospital, Aarhus, Denmark; Elective Surgery Centre, Regional Hospital Silkeborg, Silkeborg, Denmark; Department of Orthopaedic Surgery, Aarhus University Hospital, Aarhus, Denmark; Centre of Research in Rehabilitation, Aarhus University Hospital, Aarhus, Denmark; Department of Public Health, Aarhus University, Aarhus, Denmark

**Keywords:** Lumbar spinal fusion, Low back pain, Cognitive-behavioural therapy, Acute postsurgical pain, Mobility, Randomised controlled trial

## Abstract

**Background:**

Catastrophic thinking and fear-avoidance belief are negatively influencing severe acute pain following surgery causing delayed ambulation and discharge. We aimed to examine if a preoperative intervention of cognitive-behavioural therapy (CBT) could influence the early postsurgical outcome following lumbar spinal fusion surgery (LSF).

**Methods:**

Ninety patients undergoing LSF due to degenerative spinal disorders were randomly allocated to either the CBT group or the control group. Both groups received surgery and postoperative rehabilitation. In addition, the CBT group received a preoperative intervention focussed on pain coping using a CBT approach. Primary outcome was back pain during the first week (0–10 scale). Secondary outcomes were mobility, analgesic consumption, and length of hospitalisation. Data were retrieved using self-report questionnaires, assessments made by physical therapists and from medical records.

**Results:**

No difference between the groups’ self-reported back pain (*p* = 0.76) was detected. Independent mobility was reached by a significantly larger number of patients in the CBT group than the control group during the first three postoperative days. Analgesic consumption tended to be lower in the CBT group, whereas length of hospitalisation was unaffected by the CBT intervention.

**Conclusion:**

Participation in a preoperative CBT intervention appeared to facilitate mobility in the acute postoperative phase, despite equally high levels of self-reported acute postsurgical pain in the two groups, and a slightly lower intake of rescue analgesics in the CBT group. This may reflect an overall improved ability to cope with pain following participation in the preoperative CBT intervention.

**Trial registration:**

The study was approved by the Danish Protection Agency (2011-41-5899) and the Ethics Committee of the Central Denmark Region (M-20110047). The trial was registered in Current Controlled Trials (ISRCTN42281022).

## Background

A high level of acute pain following surgery, may adversely affect short-term physical and psychological recovery, thereby delaying mobilisation and discharge from hospital [1, 2]. Acute pain is furthermore a risk factor for the development of chronic postsurgical pain, which has a negative long-term impact on the patient’s life, and potentially imposes a significant burden on the healthcare sector and society [1, 3, 4]. The available knowledge about acute postsurgical pain in patients undergoing lumbar spinal fusion surgery (LSF) is limited. To our knowledge, only three studies have reported on acute postsurgical pain following LSF. One Danish observational study measured pain on a 0–10 visual analogue scale (VAS) during the first 24 h after LSF, and found patients to report an average VAS score of 5 [5]. Two other studies, one comparing different analgesic methods and one comparing different surgical techniques, found patients to report pain levels below 3 on a VAS scale. One of these studies measured pain on the first three postoperative days [6], and the other on the third postoperative day and on the day of discharge, respectively [7].

The experience of postsurgical pain is reinforced by psychological characteristics like fear-avoidance belief and catastrophic thinking [8, 9]. These negative characteristics seem to be susceptible to change through cognitive-behavioural therapy (CBT) with subsequent improvements in pain and function [10]. In the case of LSF, two studies have examined the effect of using CBT in the postoperative rehabilitation, overall reporting a positive effect of CBT in the long run [11, 12]. However, as the CBT intervention was offered several weeks after surgery in both studies, the ability of CBT to influence the acute postoperative phase is unclear. The effect of a preoperative rehabilitation intervention on early postoperative outcomes after LSF was examined in a study published by Nielsen et al. in 2010 [13]. In their study, a prehabilitation intervention with presurgical training and information, but *not* CBT, was found to be more effective than standard care in terms of improving the patients’ mobility (e.g. walking, stair climbing, daily function on ward) and decreasing length of hospitalisation [13]. Additionally, acute postsurgical pain was measured on a VAS scale (0–100) on the day of discharge, where no difference between groups was found. In 2015, the authors of this article published an RCT investigating the effect of a preoperative CBT intervention on LSF patients [14]. This RCT revealed that the CBT intervention was superior to usual care in terms of improvement in disability and quality of life three months after surgery. We therefore found it relevant to explore whether the CBT intervention could also have an effect on the patient’s pain and mobility already in the early postoperative phase. Thus, the aim of the present study was to examine whether a preoperative CBT intervention could influence acute postsurgical pain, mobility, analgesic intake and length of hospitalisation in patients undergoing LSF. This aim is in accordance with the previously published study protocol (ISRCTN42281022) [15].

## Methods

The patients were recruited consecutively from the orthopaedic departments at two Danish hospitals in the period October 2011 to June 2013. Patients who fulfilled the inclusion criteria were informed about the study by the nurses in the ambulatory. The inclusion criteria were 1) a primary diagnosis of degenerative disc disease, stenosis or spondylolisthesis grade 1–2 (assessed by the operating surgeon); 2) fusion of maximum three levels, 3) age between 18 and 64 years, and 4) competence in the Danish language. Patients were excluded if the waiting time for surgery was less than 4 weeks, a driving distance of more than 80 km from the hospital, or in case of psychiatric, inflammatory or malignant disease. Eligible patients consenting to participation were assigned by computer-generated block randomisation (by hospital) on a 1:2 ratio to receive either standard treatment (surgery and postoperative rehabilitation) or standard treatment plus a preoperative CBT intervention. The 1:2 ratio was applied to enable group sessions in the intervention group. Due to the nature of the intervention, the patients could not be blinded to treatment allocation. The trial was registered in Current Controlled Trials (ISRCTN42281022, please see http://www.isrctn.com/search?q=ISRCTN42281022).

### Interventions

The full details of the interventions are described in a previous publication [15]. Briefly, the interventions were as follows:

- Control group
  Patients in the control group received the standard course of treatment, which includes preoperative information about the upcoming operation, the anaesthetic procedure, medication, the postoperative rehabilitation and physical restrictions following surgery. Information was given by the operating surgeon, nurses and therapists. The surgical technique used was at the discretion of the surgeon.
- Cognitive-behavioural group
  In addition to the standard course of treatment, the patients were to participate in four 3-h group-sessions. The main topics covered were the interaction of cognition and pain perception, coping strategies, pacing principles, ergonomic directions, return to work, and details about the surgical procedure. The intervention was managed by a multidisciplinary team consisting of a psychologist, an occupational therapist, a physical therapist, a spine surgeon, a social worker and a previously operated patient. Each treatment session was standardised although some flexibility was allowed to meet the participants’ needs. To ensure uniformity of the sessions during the project period a staff manual was handed out to supplement the treatment sessions, and furthermore the primary investigator met with the staff at regular intervals. Compliance with the intervention was defined a priori as attendance at half of the sessions as a minimum.
- Outcomes
  The patients’ demographic characteristics were collected from the hospitals’ medical records system and from self-report questionnaires.
  The primary outcome was the severity of back pain during the first postoperative week. The patients reported their average daily pain in a diary using a numeric rating scale (NRS) of 0–10 [16], and the median of postoperative days 1–7 was calculated, in accordance with the recommendations from the International Association for the Study of Pain (IASP) [1].
  Secondary outcomes included postoperative mobility, consumption of rescue analgesics (i.e., analgesic consumption beyond the standardised analgesic protocol), and length of hospitalisation. Mobility was measured on the first three postoperative days using the Cumulated Ambulation Score (CAS) [17]. The CAS measures the level of mobility in the three activities getting in and out of bed, sit-to-stand from a chair, and walking. Each activity was assessed daily on a three-point scale from 0–2 (0 = Not able to, 1 = Able to, with assistance, 2 = Able to safely, without assistance). The assessment was performed by the physical therapist attending the patient at the ward.
  The information on analgesic consumption was retrieved from the medical records system. Due to great variability in analgesic medications and dosages, we converted these data into daily morphine-equivalent doses to enable comparison between the groups [18].
  Data on number of days of hospitalisation were retrieved from the medical records system.
- Statistical methods
  The analysis was based on intention-to-treat. All data were entered twice into EpiData version 3.1, and any divergence was corrected according to original data. STATA version 13.0 (Stata Corp, College Station, TX) was used for statistical evaluation. Due to the non-parametrical nature of the primary outcome, postsurgical back pain (0–10 scale) the difference between group medians (with 25th and 75th percentiles) was compared using the Wilcoxon rank sum test. A difference of two NRS-points was considered a clinically relevant difference between groups. Our sample size calculation was, however, based on the Oswestry Disability Index (ODI), and not the numeric pain rating scale, as the power was set to show an effect on functional disability at the one-year follow-up. These data have been presented in a previous publication, in accordance with the published study protocol for this trial [15]. For comparison of secondary outcomes (mobility, medication and hospitalisation), which were all of a non-parametrical nature, the Wilcoxon rank sum test or the chi squared statistic were used as appropriate.

## Results

### Patient recruitment and flow

**Fig. 1:**
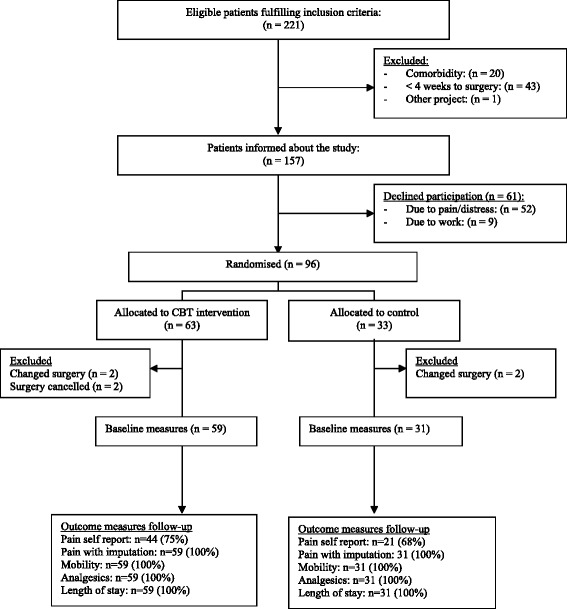
Patient recruitment, allocation and follow-up

During the inclusion period from 1 October 2011 to 1 July 2013 a total of 221 patients fulfilled the inclusion criteria. After applying the exclusion criteria 157 patients remained, and of these 96 agreed to participate (Fig. 1). Most patients declined participation due to severe pain making them unable to drive to and from the hospital in case of allocation to the CBT group, and some declined because they could not take time off work. The majority of the patients (81 of 96) were recruited from one of the two hospitals (the regional hospital). Sixty-three patients were allocated to the intervention group, and 33 to the control group. Six patients were excluded (4 in the CBT group) at the time of surgery due to change of surgical technique or cancellation of surgery. The median number of days from baseline measurement to surgery was 42.5 days (range 26–210 days) for the group as a whole. Table 1 shows the patients’ baseline characteristics.

**Table 1:** Baseline patient data

Patient characteristics	CBT group	Control group
	(*n* = 59)	(*n* = 31)
Male gender	23 (39)	16 (52)
Age (year ± SD)	51.4 (9.2)	47.7 (8.9)
Smoking n (%)	20 (32)	10 (30)
Working status		
Employed Unemployed Disability pension Early retirement	32 (54) 11 (19) 9 (15) 7 (12)	15 (48) 11 (36) 5 (16) 0 (0)
Diagnosis		
Spondylolisthesis	16 (27)	7 (23)
Degenerative disc disease	43 (73)	24 (77)
Surgical procedures		
PLF	41 (69)	12 (39)
TLIF	17 (29)	19 (61)
Uninstrumented	1 (2)	0 (0)
Previous spine surgery		
Spondylodesis	2 (3)	1 (3)
Decompression	7 (11)	1 (3)
Fusion levels		
One	36 (62)	20 (69)
Two	19 (32)	8 (27)
Three	4 (7)	3 (10)
Disability (ODI)		
Mean (SD)	40.7 (13.2)	40.8 (15)
Pain (LBPRS)		
Back,median (25;75 percentile)	7.0 (5.3;8.0)	7.2 (6.0;8.0)
Leg, median (25;75 percentile)	6.3 (4.3;7.7)	6.3 (3.7;8.3)
Quality of life (EQ-5D)	0.655 (0.389;0.723)	0.627 (0.356;0.723)

Numbers are presented as *n* (%) unless otherwise stated

*EQ-5D* EuroQol 5 Dimensions, *LBPRS* low back pain rating scale, *ODI* oswestry disability index, *PLF* posterolateral fusion, *TLIF* transforaminal lumbar interbody fusion

### Clinical outcomes

The results for all outcome measures are presented in Table 2.

**Table 2:** Pain, mobility, analgesic use, and length of stay after fusion in CBT and control group

Outcome Reported as median (25th; 75th percentiles) unless stated otherwise	CBT group	N	Control group	N	*P*-value
Back pain^a^	5.4 (4.0;6.5)	44	5.3 (4.0;6.1)	21	0.74
Mobility on day 3, n (%)^b^		59		31	
Walk	43 (73)		15 (48)		0.02
Rise and sit from a chair	58 (98)		26 (84)		0.017
Get in and out of bed	58 (98)		26 (84)		0.017
Analgesic use		59		31	
Morphine equivalents	142.5 (70;275)		196.8 (145;345)		0.23
Hospitalisation		59		31	
Number of days	5 (4;6)		4 (4;6)		0.46

^a^Pain: measured using numeric rating scale (0-10/best-worst)

^b^The numbers indicate the number (%) of patients being able to carry out the activity without support on the third postoperative day

For the primary outcome the CBT group reported a median back pain severity of 5.6 (range 1.7–10.0), which was no different from that of the control group of 5.3 (range 1.1–7.7) (*P* = 0.73). Due to a non-response rate of 28 % on the pain diaries, we chose to perform a sensitivity analysis where missing data on back pain were imputed with data retrieved from the medical records. This reduced the median scores slightly, although non-significantly, with a median score of 4.9 (range 1.7–10) and 4.9 (range 1.1–7.8) for the CBT group and the control group, respectively.

**Fig. 2:**
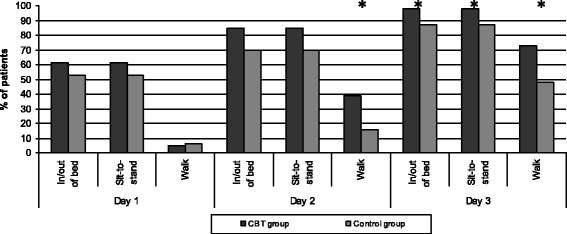
Independent mobility the first 3 days after lumbar fusion surgery. Columns represent the percentage of patients in each group who are able to manage the given activity without any assistance from a person or an aid. CBT = cognitive-behavioural therapy. * = *P* < 0.05

With regards to postoperative mobility patients in the CBT group performed superiorly on the measured activities during the first 3 postoperative days (Fig. 2). Thus, independent walking ability was achieved by 39 % (*n* = 23) versus 16 % (*n* = 5) (*P* < 0.026) on day 2, and by 73 % (*n *= 43) versus 48 % (*n* = 15) of patients on day 3 (*P* < 0.02). For the activities *getting in and out of bed* and *getting up from a chair* the CBT group correspondingly performed better on all three days of assessment, with the difference being significant on day 3 (Fig. 2).

**Fig. 3:**
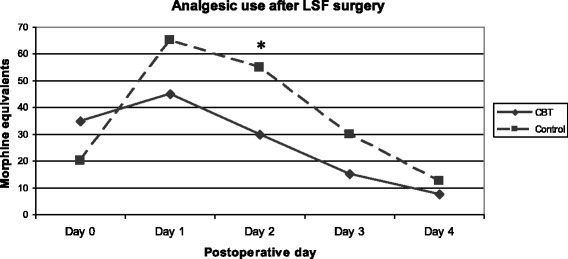
Use of analgesics during the first 5 postoperative days following lumbar spinal fusion surgery. Units in morphine equivalent dose. Graph illustrating median values. Day 0 = the day of surgery; CBT = cognitive behavioural therapy group. *P < 0.05

In terms of analgesic consumption, a lower consumption was observed in the CBT group, although the difference between groups was only significant on postoperative day 2 (Fig. 3).

The length of stay was comparable between the groups with a median number of days of five (range 3–9) in the CBT group and four (range 3–10) in the control group.

### Compliance with intervention

Of the 49 patients who complied with the intervention 15 patients attended all 4 sessions, 21 attended 3 sessions and 13 attended 2 sessions. For the ten patients that did not comply (i.e., attended only 1 or no sessions) various reasons for non-compliance were given, e.g., could not take time off as expected (*n* = 3), driving to and from hospital caused too much pain (*n* = 2), on maternity leave prior to surgery (*n* = 1), serious illness of close relative (*n* = 1) and other personal reasons (*n* = 3). All patients remained in the analysis, regardless of their compliance with the intervention.

## Discussion

The purpose of our study was to investigate whether a preoperative CBT intervention had a positive effect on acute postsurgical pain, mobility, use of analgesics, and length of hospitalisation. We did not find CBT to be superior in terms of pain reduction. In fact, both groups reported a moderate to severe level of back pain (>5 NRS points) postoperatively, which is comparable to the findings of the Danish observational study previously described [5]. These pain levels are high compared with the results of the other studies, generally reporting postoperative pain levels below 3 points [6, 7, 13]. These differences may be due to different methods for reporting acute postsurgical pain, as these studies reported pain using VAS scales where we used the NRS scale. However, there seems to be little difference between the two scales, as the literature finds VAS and NRS scores to correspond well and also finds them equally responsive [16, 19]. Also, the differences could be due to variability in the diagnosis of the study populations, as two of the studies included only patients with spondylolisthesis grade I and II [6, 7]. The third study did not report specifically on the patients' diagnosis (“degenerative disease”) [13].

An important finding of the present study was the significantly superior achievements on mobility, seen in the CBT group. Despite the fact that patients in the two groups experienced the same severity of acute postsurgical pain, the patients in the CBT group performed better on the assessed activities, even with a lower requirement for rescue analgesics. These results correspond well with the previously published findings on disability for this study, where the CBT group reported a significantly larger reduction on the Oswestry Disability Index (*P* = 0.003) already 3 months after LSF surgery, even though self-reported back pain was similar to that of the control group [14].

In terms of length of hospitalisation, patients in the CBT group were not discharged from hospital earlier than patients in the control group. As suggested by Kehlet et al. [20] this may indicate that factors like established ward routines and the staff and the patient’s expectations have a greater say for when patients are discharged than the patient’s actual condition itself. Similarly, in the prehabilitation study by Nielsen et al., routines in discharge procedures caused a delay between the achievement of recovery milestones and the time of discharge [13].

Our sample size calculation was based on the Oswestry Disability Index (ODI) and not the NRS, as the power was set to show an effect on functional disability at the 1-year follow-up. These data have been presented in a previous publication in accordance with the published study protocol for this trial [15]. We therefore cannot rule out that our study was insufficiently powered to demonstrate differences in pain. However, even with the low response of patients ( 44 from the CBT group and 21 from the control group) it allows for the detection of a difference between the groups of 2.0 NRS units (SD 2.0) at a significance level of 0.05 with a power of 80 %. Hence, a more powerful design would probably not have affected the overall conclusions regarding pain.

We included both spondylolisthesis and disc degeneration patients in the study to mimic daily clinical practice and to increase the external validity of the study. By random, the two posterior surgery procedures (PLF and TLIF) were unevenly distributed in the two groups. As the TLIF is a procedure with longer surgery time than the PLF, a difference could perhaps have been expected in this early recovery phase. However, in the present study this was not the case, as patients undergoing TLIF reported lower pain levels during hospitalisation in both the CBT group and the control group.

A limitation to our study was the moderate response rate of 72 % for the pain diaries. However, a non-responder analysis revealed no significant differences between non-responders and responders regarding their baseline characteristics, e.g. gender (*P* = 0.69), age (*P* = 0.25), ODI score (*P* = 0.97) and pain severity (*P* = 0.83).

Another limitation study pertains to the issue of blinding, an almost unavoidable limitation of studies investigating complex interventions. Thus, although the physiotherapists assessing mobility (CAS score) were not informed about the patients’ group assignment, a few of the patients accidentally revealed this. This may of course have had an influence in favour of the CBT group for those patients whose group assignment was known.

## Conclusion

We believe that this study adds important knowledge about the early postoperative period following LSF. Although the preoperative CBT intervention did not influence acute postsurgical pain, the intervention had a positive effect on the patients’ ability to cope with pain as evidenced by earlier achievement of independent mobility and less use of analgesics in the intervention group. With reference to the previously published results from this trial, showing an overall positive effect of the CBT intervention, we therefore recommend that a CBT intervention is offered prior to LSF surgery. We further find it relevant to investigate whether a change in ward procedures would enable a faster discharge for those with independent mobility already on postoperative day two or three.

## Abbreviations

CAS: cumulated ambulation score
CBT: cognitive-behavioural therapy
EQ-5D: EuroQol 5 dimensions
LSF: lumbar spinal fusion
NRS: numeric rating scale
ODI: oswestry disability index
VAS: visual analogue scale
